# SLMSuite: a suite of algorithms for segmenting genomic profiles

**DOI:** 10.1186/s12859-017-1734-5

**Published:** 2017-06-28

**Authors:** Valerio Orlandini, Aldesia Provenzano, Sabrina Giglio, Alberto Magi

**Affiliations:** 1 Medical Genetics Unit, Meyer Children’s University Hospital, Florence, Italy; 2 Department of Experimental and Clinical Medicine, University of Florence, Viale Pieraccini 6, Florence, 50139 Italy

**Keywords:** Software, Genomics, Bioinformatics, SLM

## Abstract

**Background:**

The identification of copy number variants (CNVs) is essential to study human genetic variation and to understand the genetic basis of mendelian disorders and cancers. At present, genome-wide detection of CNVs can be achieved using microarray or second generation sequencing (SGS) data. Although these technologies are very different, the genomic profiles that they generate are mathematically very similar and consist of noisy signals in which a decrease or increase of consecutive data represent deletions or duplication of DNA. In this framework, the most important step of the analysis consists of segmenting genomic profiles for the identification of the boundaries of genomic regions with increased or decreased signal.

**Results:**

Here we introduce SLMSuite, a collection of algorithms, based on shifting level models (SLM), to segment genomic profiles from array and SGS experiments. The SLM algorithms take as input the log-transformed genomic profiles from SGS or microarray experiments and output segmentation results. We apply our method to the analysis of synthetic genomic profiles and real whole genome sequencing data and we demonstrate that it outperforms the state of the art circular binary segmentation algorithm in terms of sensitivity, specificity and computational speed.

**Conclusion:**

The SLMSuite contains an R library with the segmentation methods and three wrappers that allow to use them in Python, Ruby and C++. SLMSuite is freely available at https://sourceforge.net/projects/slmsuite.

**Electronic supplementary material:**

The online version of this article (doi:10.1186/s12859-017-1734-5) contains supplementary material, which is available to authorized users.

## Background

Copy number variants (CNVs) are DNA segments larger than 50 bp [1] that are present at a variable number of copies with respect to a reference genome. CNVs represent one of the main sources of genetic diversity in humans [2], and some of them have been demonstrated to be associated with many disease states such as cancer, autoimmune diseases, cardiovascular disease, and Alzheimer and Parkinson diseases [3].

At present, the identification of CNVs, at a genome-wide level, can be performed by using array-based comparative genomic hybridization (aCGH), SNP arrays and second generation sequencing (SGS). Although the experimental strategies at the base of these technologies are very different, the genomic signals that they generate for CNVs identification are mathematically very similar.

Read count (RC) [4] data for SGS and log2-ratio for array platforms are noisy signals of spatially ordered data in which deletions or duplications are identified as a decrease or increase of the signal. From a computational point of view the fundamental step in the identification of CNVs consists of segmenting RC/log2-ratio for identifying the boundaries and estimating the mean level of these increase or decrease of the signal. While the use of SGS data becomes routine and third generation sequencing is emerging, the availability of very accurate and fast segmentation algorithms is becoming fundamental.

In the last few years we developed a class of algorithms, based on shifting level models (SLM), that allow to segment with high accuracy genomic profiles. The first SLM algorithm [5] was developed for analyzing log2-ratio data from CGH-array, the multivariate version, JointSLM [6] was written for the joint segmentation of multiple RC signals, while the heterogeneous version, heterogeneous shifting levels model (HSLM) [7] was properly tailored for segmenting spatially sparse data from whole-exome sequencing (WES) experiments.

Here we present a suite of segmentation methods, named SLMSuite, that contains the SLM and HSLM algorithms for the analysis of genomic profiles from microarray and SGS data. By using synthetic and real genomic profiles we demonstrate that our algorithm outperforms the circular binary segmentation [8] (CBS) method in terms of both sensitivity and specificity.

## Implementation

The SLMSuite is developed as a package (SLMSeg) for the statistical environment R and includes two main functions SLM and HSLM. The two functions take as input the Log2-Ratio data and starting parameters and give as output the results of the segmentation performed by SLM and HSLM respectively.

Along the R library, there are three wrappers that, using specific libraries, allows one to use the two R functions directly in Python, Ruby and C++. The wrappers call the original R functions and have in common that they provide a class or a module (SLMSeg) that is able to store the parameters and the data and to read the signal information directly from a file.

SLMSuite is freely available at https://sourceforge.net/projects/slmsuite. Once installed, a comprehensive manual can be found inside the doc folder.

## Results

### Shifting level model algorithms

**Fig. 1:**
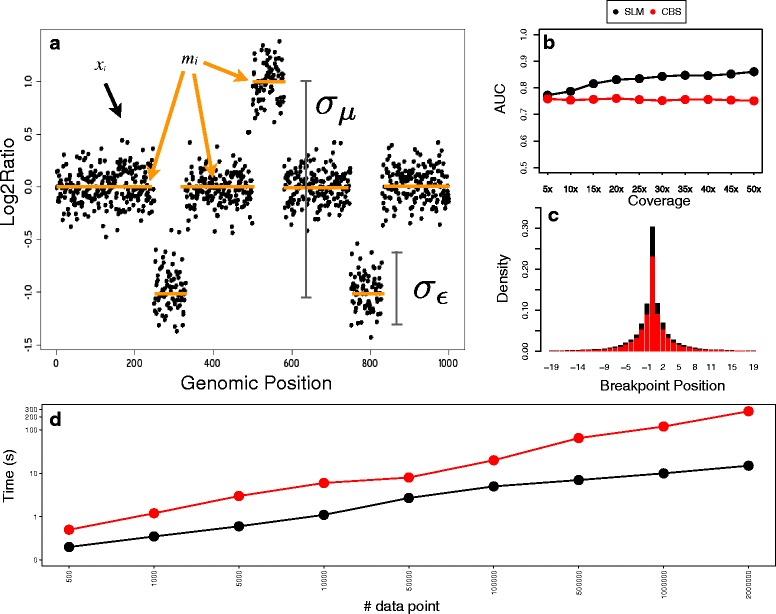
Performance comparison between SLM and CBS algorithms on simulated data. Panel **a** shows how genomic profiles are modeled by SLM. *Black dots* are the observations *x*_*i*_, orange segments are the unobserved mean levels *m*_*i*_ and *vertical black bars* represent the ranges of $\sigma _{m}^{2}$ and $\sigma ^{2}_{\epsilon }$. Panel **b** reports the area under the receiver operating characteristic curve (AUC) as a function of sequencing coverage for SML and CBS. Panels **c** summarizes the performance of SLM and CBS algorithms in the detection of the correct breakpoint position, while panel d reports the computational speed of the two methods in segmenting genomic profiles made of different number of data points (analyses were performed on a 2.5 GHz Intel Core i5 with 8 Gb of RAM). *Black dots* represent SLM, while red ones CBS. On the x axis of panel c is reported the distance between the predicted and the correct breakpoint position, while on the y axis is reported the fraction of breakpoints predicted at a given distance from the correct position

SLMs [5] model noisy sequential processes *x*=(*x*_1_,..,*x*_*i*_,..,*x*_*N*_) that show sudden shifts in the mean as the sum of two independent stochastic processes:

1 $$ x_{i}=m_{i}+\epsilon_{i},  $$

2 $$ m_{i}=(1-z_{i-1})\cdot m_{i-1}+z_{i-1}\cdot(\mu+\delta_{i}).  $$

where *m*_*i*_ is the unobserved mean level that follows a normal distribution with mean *μ* and variance $\sigma ^{2}_{m}$ (*m*_*i*_∼*N*(*μ*,*σ* ^2^_*m*_)) and *ε*_*i*_ is a normally distributed white noise with variance $\sigma ^{2}_{\epsilon }$ ($\epsilon _{i} \sim N(0, \sigma ^{2}_{\epsilon })$, Fig. 1a).

The process *m*_*i*_ changes its value independently of *m*_*i*−1_ and is controlled by the process *z*_*i*_ : when *z*_*i*−1_=0, *m*_*i*_ is the same as *m*_*i*−1_ and when *z*_*i*−1_=1, *m*_*i*_ is incremented by the normal random variable *δ*_*i*_ ($\delta _{i} \sim N(0,\sigma _{m}^{2})$). *z*_1_,*z*_2_,… are independent and identically distributed random variables taking the values 0 or 1 with probabilities *η*=*P* *r*(*z*_*i*_=1) or 1−*η*=*P* *r*(*z*_*i*_=0), respectively. SLM is a particular class of hidden markov models (HMM) and thanks to this property we developed a powerful algorithm, based on classical HMM parameter estimation methods (Baum and Welch and Viterbi algorithms) that is able to segment aCGH signals for the identification of deletions and duplications.

In [7] we improved the SLM by changing its architecture from a homogeneous to heterogeneous HMM (HSLM) for segmenting spatially sparse data like RC from WES experiments.

In order to take into account genomic distance between adjacent coding regions of the genome we incorporated the genomic distance in the transition matrix of the SLM by defining the probability *P* *r*(*z*_*i*_=1) in the following:

3 $$ Pr\left(z_{i}=1\right)=\eta(d_{i})= \theta+\left((1-\theta)\cdot \exp\left[\frac{log(\theta)}{\frac{d_{i}}{d_{Norm}}}\right]\right)   $$

where *η*(*d*_*i*_) is the probability of random variables *z*_*i*_ to be equal to 1, *θ* is a constant parameter, *d*_*i*_ is the distance between the *i* ^*th*^ and (*i*−1)^*th*^ targeted region and *d*_*Norm*_ is the distance normalization parameter. Equation 3 defines the dependence between the probability *P* *r*(*z*_*i*_=1) and the genomic distance between adjacent targeted regions *d*_*i*_: the larger genomic distance and the larger *P* *r*(*z*_*i*_=1) and consequently the larger the probability to jump between two mean levels *m*_*i*_.

The constant parameter *θ* can be seen as the baseline probability of random variables *z*_*i*_ to take value 1 while the *d*_*Norm*_ parameter modulates the genomic distance at which the probability *P* *r*(*z*_*i*_=1) begins to grow: for distances much smaller than *d*_*Norm*_ the probability *P* *r*(*z*_*i*_=1)=*θ*, while when *d*_*i*_ is larger than *d*_*Norm*_ the probability *P* *r*(*z*_*i*_=1) grows until reaching the value 1. The *d*_*Norm*_ parameter is fundamental for modulating the resolution of HSLM algorithm: the smaller the value of DNorm the larger the probability to jump from one state to another and the higher its ability to detect small genomic events. However, small values of *d*_*Norm*_ also increase the total number of FP events detected [7].

### SLM vs CBS on synthetic and real data

To demonstrate the power of SLM algorithm in detecting CNVs of different size, we performed an intensive simulation based on synthetic data and we compared its performance to the most widely used and cited algorithm (CBS) for segmenting genomic profiles from aCGH and SGS experiments.

Synthetic genomic profiles were generated from the RC data (normalized as in [4]) of three whole-genome sequencing (WGS) experiments (NA12878, NA12891 and NA12892) selected from the Illumina Platinum collection (downloaded at ftp://ftp.sra.ebi.ac.uk/vol1/ERA172/ERA172924). The Illumina platinum collection comprises the WGS data of 17 members of the Coriell CEPH/UTAH 1463 family sequenced with the Illumina HiSeq 2000 platform at a coverage of 50x. The BAM files of the three WGS experiments were processed, sorted and filtered (discarding MQ ≤ 10) with SAMtools and PCR duplicates were removed with Picard MarkDuplicates (http://picard.sourceforge.net). In order to simulate WGS data at different coverages, each 50x experiment was downsampled with SAMtools to obtain coverages at 5x, 10x, 15x, 20x, 25x, 30x, 35x, 40x, 45x and 50x.

The three genomes used in this analysis were previously characterized by McCarroll et al. [9] using an hybrid SNP-array platform (Affymetrix SNP 6.0) that simultaneously interrogates 906,600 SNPs and copy number at 1.8 million genomic locations. McCarroll et al. [9] used this SNP-array platform on 270 HapMap samples to construct an accurate map of the boundaries and the integer copy number level of the genomic regions affected by CNVs in each individual. The boundaries of each CNV were determined by means of an Hidden Markov model and the estimation of integer copy number level was performed by means of quantitative PCR.

For each BAM file (three individuals at ten different coverages), RC data were calculated, normalized (for GC-content and mappability as in [4]) and log2 transformed for four different window size: 100, 200, 500 and 1000 bp. Synthetic genomic profiles were simulated with the following recipe:

- 2-copies regions were simulated by sampling (10000-N) RC data from genomic regions previously predicted as 2-copies by McCarroll *et al* for the NA12878, NA12891 and NA12892 samples.
- 1-copy (3-copies) regions were simulated by sampling N RC data from regions previously predicted as 1-copy (3-copies) for NA12878, NA12891 and NA12892 samples.

We performed simulations with N =1, 2, 3, 4, 5, 10, 20, 30, 40, 50, 100, 200, 300, 400, 500. For each N, window size and coverage we generated 1000 synthetic genomic profiles.

To evaluate the capability of our algorithm in identifying CNVs at the boundaries (breakpoints detection), we calculated the receiver operating characteristic (ROC) curve as in [10] and we compared SLM performance to that of CBS [8].

Moreover, to test the ability of the two segmentation algorithms in correctly identifying the exact CNV breakpoint, we calculated the distance (in windows) between the correct and the predicted breakpoint position.

Figure 1b-c and Additional file 1: Figures S1 and S2 clearly show that SLM outperforms CBS in terms of both sensitivity and specificity for all the noise levels we simulated and that is capable to detect the exact breakpoint with higher accuracy. Remarkably, while CBS gives similar results for all the noise levels we simulated, SLM accuracy increases at the increasing of coverages and window sizes, in particular for coverages smaller than 20x. Surprisingly, for low coverage (5x) and small window size (100 bp) CBS obtains AUC values higher than SLM, and this can be ascribed to the higher number of FP detected by SLM. However, the optimal window size scales inversely with the coverage, resulting in 500 bp for 5x experiments [4]. In this range SLM clearly outperform CBS.

As a further step, we assessed the capability of SLM to discover CNVs by exploiting the method reported in [6 *,* 7]: a detected alteration is considered a true positive if there is any overlap any synthetic altered region, while it is considered a false positive if there is no overlap with any synthetic altered region (Additional file 1: Figure S3).

SLM obtain higher resolution (the capability of identifying CNVs made of small number of windows, Additional file 1: Figure S3) than CBS with a computational speed much larger than that required by the other state of the art segmentation algorithm (Fig. 1d). In particular, for datasets made of large number of windows (≥50000) SLM was able to segment genomic profiles in less than 10 seconds while CBS scaled up in the order of minutes. This result is of great relevance for the analysis of high coverage whole genome sequencing data with small window size (100 bp) that generate genomic profiles up to 2.5 millions of RC data points.

Finally, in order to show the potentialities of our SLM algorithm in segmenting real genomic profiles, we applied it to the analysis of the Illumina Platinum WGS experiments of the three individuals described above (NA12878, NA12891 and NA12892) and we compared the results with those obtained by CBS.

To compare the performance of the two segmentation algorithms in identifying CNVs, we calculated precision and recall rates by using the McCarroll dataset as reference set: precision was calculated as the the ratio between the number of correctly detected CNVs and the total number of CNVs detected by each algorithm, while recall was calculated as the ratio between the number of correctly detected CNVs and the total number of CNVs in the McCarroll dataset. Since the capability of detecting genomic regions involved in CNVs is influenced by the length of the event, we distinguished three classes of variants: Small (*l* *e* *n* *g* *t* *h*<20*K* *b*), Medium (*l* *e* *n* *g* *t* *h*≥20*K* *b* and <100*K* *b*) and Large (*l* *e* *n* *g* *t* *h*≥100*K* *b*).

**Fig. 2:**
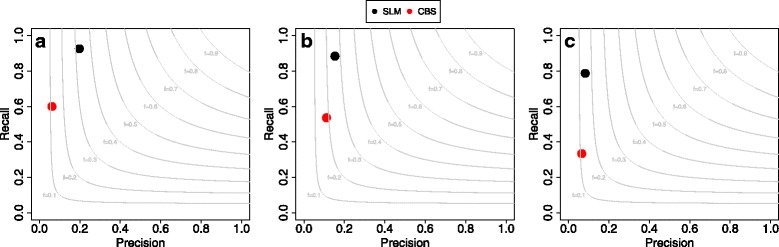
Performance comparison between SLM and CBS algorithms on real data. Summary of the results obtained by SLM and CBS on the analysis of the three platinum genomes. In the three panels are reported the precision-recall plots of the comparison between the CNV events detected by SLM and CBS and the CNVs previously reported by McCarroll et al. Light grey curves represent F-measure levels (harmonic mean of precision and recall). Panel **a** report the results for large, panel **b** for medium CNVs and panel **c** for small CNVs

The results of these analyses are reported in Fig. 2 and clearly demonstrate that our algorithm outperform CBS in terms of both precision and recall for all the three size classes.

## Conclusion

Segmentation of genomic profiles obtained from aCGH, SNP-arrays, WGS and whole-exome sequencing experiments has been demonstrated to be the key step for the accurate detection of genomic regions involved in CNVs.

The availability of powerful segmentation algorithms is fundamental for the improvement of existing tools and for the development of novel computational methods for CNVs discovery. In this work we demonstrate the computational power and accuracy of SLM based algorithms with respect to the state of the art CBS method and we present a novel software package that contains all the SLM algorithms.

Thanks to the SLMSuite, all the SLM algorithms can be easily integrated into existing or novel pipelines written in different programming languages.

## Additional file

Additional file 1 Supplementary figures. The pdf file contains **Figures S1-S3.** (PDF 86.9 kb)

## Abbreviations

aCGH: Array-based comparative genomic hybridization
CBS: Circular binary segmentation HMM: Hidden markov models
HSLM: Heterogeneous shifting levele model RC: Read count
SGS: Second generation sequencing
SLM: Shifting level models
WGS: Whole genome sequencing
