# Supplementary material for: SLMSuite: a suite of algorithms for segmenting genomic profiles
Source: BMC Bioinformatics. 2017 Jun 28;18:321. doi: 10.1186/s12859-017-1734-5 (PMC5490196; doi:10.1186/s12859-017-1734-5)
Supplement: Additional file 1 — Supplementary figures. The pdf file contains Figures S1-S3. (PDF 86.9 kb) [file 12859_2017_1734_MOESM1_ESM.pdf]

Supplemental Material to: SLMSuite: a suite of algorithms for segmenting genomic profiles.

Valerio Orlandinii[1], Aldesia Provenzano[1], Sabrina Giglio[1], Alberto Magi[2].

[1]Medical Genetics Unit, Meyer Children's University Hospital Florence, Italy ; Department of Clinical and Experimental Biomedical Sciences, University of Florence Florence, Italy. [2]Department of Experimental and Clinical Medicine, University of Florence, Florence, Italy,

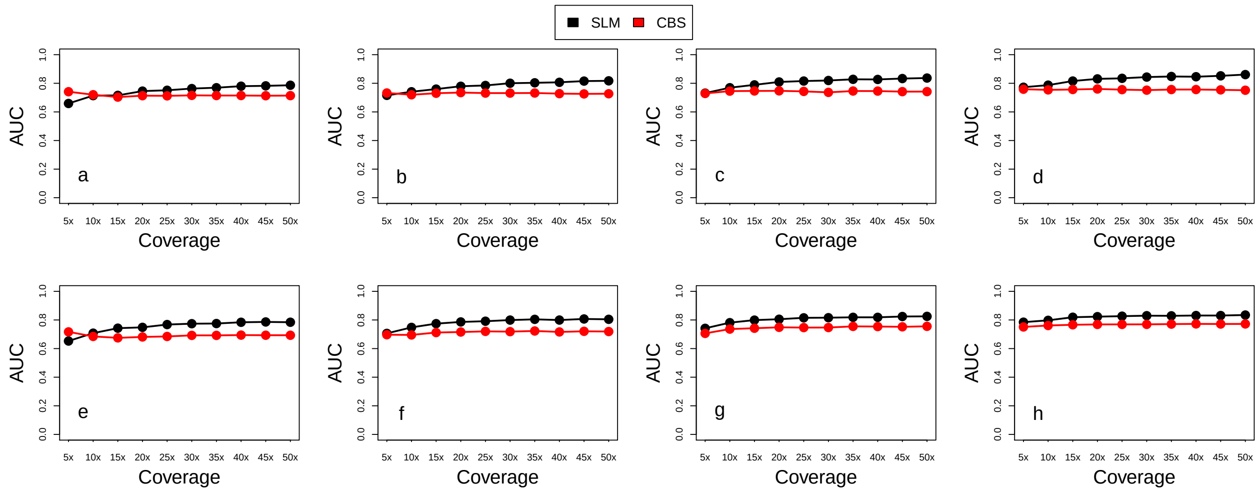

Supplementary Figure 1: AUC for SLM and CBS methods. Each panel of the figure reports the area under the receiver operating characteristic curve (AUC) as a function of sequencing coverage. Panels a-d report the results for simulated duplications, while panels e-h for simulated deletions. The analyses were performed for different window sizes: 100 bp (a, e), 200 bp (b, f), 500 bp (c, g) and 1000 bp (d, h).

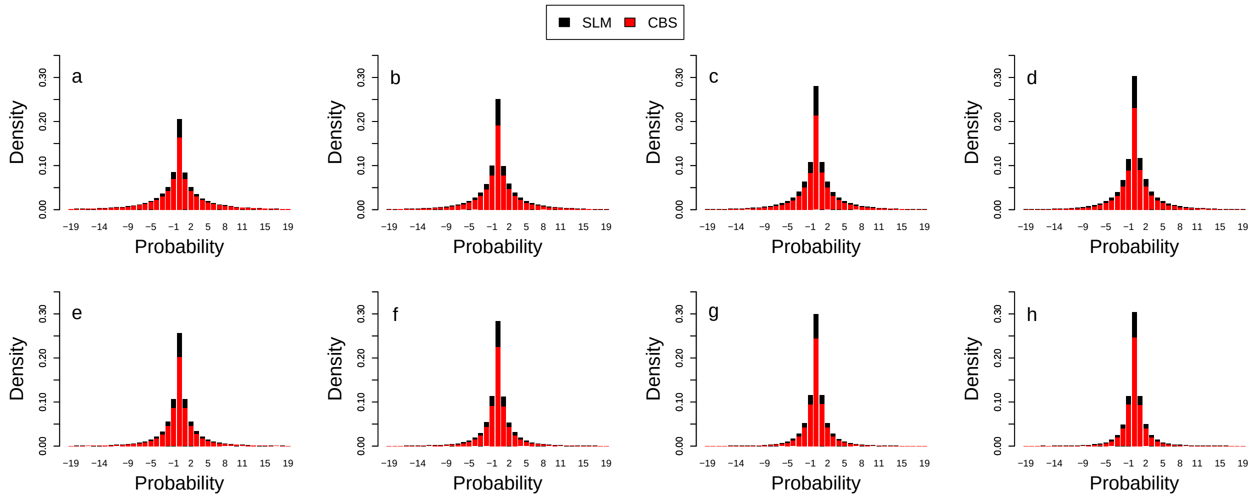

Supplementary Figure 2: Breakpoints detection accuracy. Each panel of the figure summarizes the performance of SLM and CBS algorithms in the detection of the correct breakpoint position. On the x axis is reported the distance between the predicted and the correct position. On the y axis is reported the fraction of breakpoints predicted at a given distance from the correct position. Panels a-d report the results for simulated duplications, while panels e-h for simulated deletions. The analyses were performed for different window sizes: 100 bp (a, e), 200 bp (b, f), 500 bp (c, g) and 1000 bp (d, h).

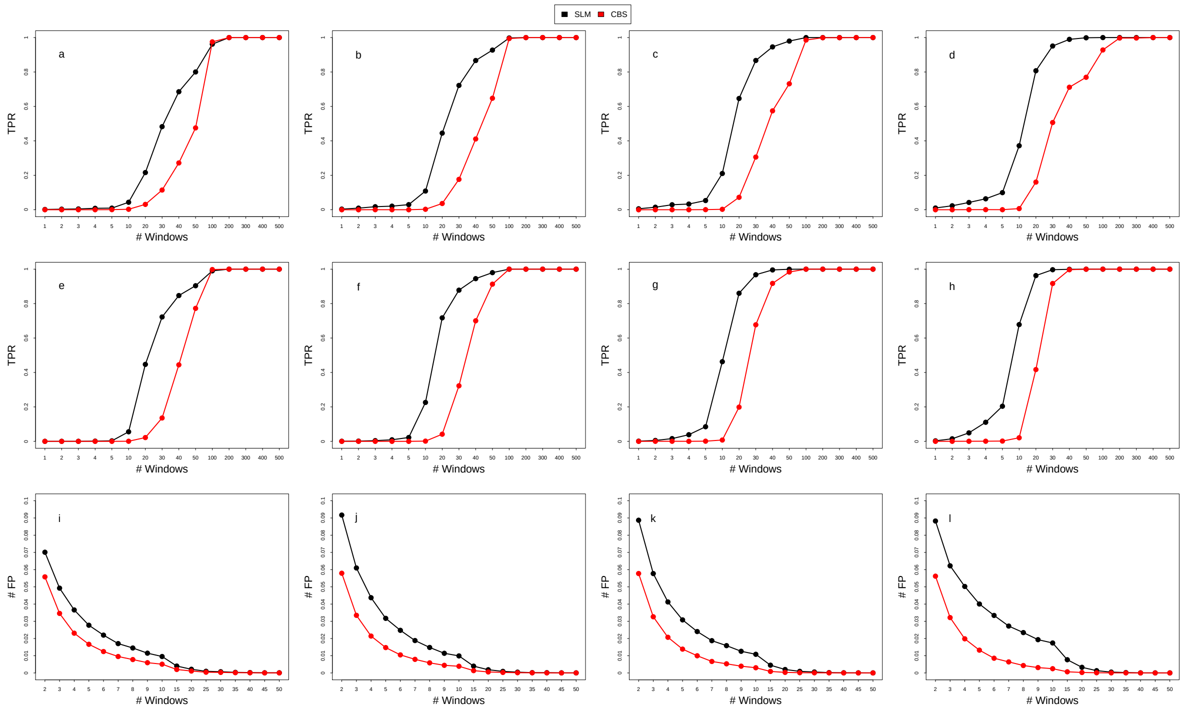

Supplementary Figure 3: True positive rate and false positive performance. The panels report TPR and FP plots for different window sizes. In panels a-d are reported the TPR for 3-copy regions, in panels e-h TPR for 1-copy regions and in panels i-l the FP. The analyses were performed for different window sizes: 100 bp (a, e, i), 200 bp (b, f, j), 500 bp (c, g, k) and 1000 bp (d, h, l). Each point is obtained by averaging across 1000 synthetic genomic profiles.
